# Supplementary material for: Different contributions of fat and lean indices to bone strength by sex
Source: PLoS One. 2024 Nov 14;19(11):e0313740. doi: 10.1371/journal.pone.0313740 (PMC11563455; doi:10.1371/journal.pone.0313740)
Supplement: Attachment — Submitted filename: Comments.pdf [file pone.0313740.s001.pdf]

Comments

The authors summarize the effects of fat and lean body mass on bone strength in men and women in this paper. It is considered to be interesting, containing new findings and elements that influence the resolution of the reader's questions. However, there are major problems with the overall structure and development of the paper. It is difficult to see the overall narrative and it is not clear what you want to argue. The explanation for the claim is insufficient. There are similar previous studies on the keywords presented. Therefore, the structure must be changed to claim novelty. Also, some results are not shown (e.g. Table 5). These indicate several major problems, which require significant modification. The comments are summarized below:

Red; Major comments   Blue; Minor comments

Abstract

Purpose

- There is insufficient logical development to show the purpose of the study. Since bone strength is related to bone density and bone quality factors, the topic of bone quality may also be necessary. In short, the title of the study is "To determine the effects of fat and lean mass on bone strength in men and women", so the abstract should provide an explanation in line with the title.

Methods

- Why are only ALM and HGS abbreviations used here? Other abbreviations are given in the text, but if they are given in the Abstract, please unify them.

Conclusion

- It is necessary to state what needs to be argued in the conclusion, rather than summarizing the results.

Introduction

- Overall, the logical development is insufficient. Please provide a clear explanation, e.g. by using headings. The use of evidence needs to be guided towards the objectives of this study.

- L59 There are no citations or references as evidence for “The World ～ evaluate obesity”.
- L61 No citation or reference as evidence is provided for “Although ～ lean mass”.
- The two places where reference [7-10] is used are Fat indices and bone density on the one hand and Fat indices and bone quality on the other. Despite being different topics, [7-10] is used. Either another previous study needs to be cited or the structure of the text needs to be revised.

Methods

Participants

- Please add the Approval number in the text.

Anthropometric measurements

- Name of measuring device for height and weight is not indicated.

Muscle strength

- In the grip strength measurement method, three measurements were carried out and then the maximum value was used. Please explain or provide evidence for this.

Bone mineral density

- For BMD measurements, the ISCD 2019 citation should be shown.

Other covariates

- "Regular exercise" needs to be specified in terms of time, intensity and frequency. Exercise affects bone density, muscle mass and strength. The type of exercise the subject is doing is also a very important factor. Particularly, body weight affects bone as a mechanical stress. Shouldn't body weight be included as a covariate?
- The type of drug is indicated in the Medication. Why is it identified as this type? Evidence is needed to show this. Are supplements included?

Results

- In all Tables, "Mean" and "Standard deviation" are indicated at the top of the table. Therefore, the notation (Mean ± SD) in Variables is probably unnecessary.
- No caption for Table 1.
- The term "bone density" is used a lot throughout the text, is this a reference to BMD? The wording is inconsistent.
- Table 5 is not listed.
- For each result, the R-value (e.g. for the measured items where significant differences are found) is unclear. It would be more comprehensible if correlation coefficients were given.
- Are all R^2 values sufficient evidence for what you are claiming or concluding in this paper? Shouldn't your views on this be described in the discussion?

Discussion

- The explanation and discussion of the results of Multiple regression is vague and difficult to understand.
- From reading the text, it is understood that the measurement of BMD was carried out on the basis of ISCD. In the multiple regression analysis, Spine, Femoral neck and Total hip are used as variables and the results (β) for these three variables are presented. However, the respective models and the discussion for Spine, Femoral neck and Total hip are not written. Therefore, the reason for using multiple regression analysis is not understood. If multiple regression is to show the relationship between the three variable results and the model, specific results and discussion should be given for this.
- It is important to use analysis methods appropriate to what should be claimed and to derive new findings.
