# Supplementary material for: Different contributions of fat and lean indices to bone strength by sex
Source: PLoS One. 2024 Nov 14;19(11):e0313740. doi: 10.1371/journal.pone.0313740 (PMC11563455; doi:10.1371/journal.pone.0313740)
Supplement: Attachment — Submitted filename: Response to reviewers.docx [file pone.0313740.s002.docx]

Reviewers' comments:

Reviewer's Responses to Questions

**Comments to the Author**

1. If the authors have adequately addressed your comments raised in a previous round of review and you feel that this manuscript is now acceptable for publication, you may indicate that here to bypass the “Comments to the Author” section, enter your conflict of interest statement in the “Confidential to Editor” section, and submit your "Accept" recommendation.

Reviewer #1: All comments have been addressed

Reviewer #2: (No Response)

**Reply:** Thank you for your comment.

2. Is the manuscript technically sound, and do the data support the conclusions?

The manuscript must describe a technically sound piece of scientific research with data that supports the conclusions. Experiments must have been conducted rigorously, with appropriate controls, replication, and sample sizes. The conclusions must be drawn appropriately based on the data presented.

Reviewer #1: Yes

Reviewer #2: Partly

**Reply:** Thank you for your comment. We have revised the abstract to reflect the same claim found on page 2, line 37.

3. Has the statistical analysis been performed appropriately and rigorously?

Reviewer #1: Yes

Reviewer #2: Yes

**Reply:** Thank you for your comment.

4. Have the authors made all data underlying the findings in their manuscript fully available?

The [PLOS Data policy](http://c05y1x9s.r.us-east-2.awstrack.me/L0/http:%2F%2Fwww.plosone.org%2Fstatic%2Fpolicies.action%23sharing/1/010f0191c6320010-e2ccbdd3-2261-43ca-a600-f0521ec8efa1-000000/zGlAlcHZrHuHjSHIxbtj81nHXNQ=175) requires authors to make all data underlying the findings described in their manuscript fully available without restriction, with rare exception (please refer to the Data Availability Statement in the manuscript PDF file). The data should be provided as part of the manuscript or its supporting information, or deposited to a public repository. For example, in addition to summary statistics, the data points behind means, medians and variance measures should be available. If there are restrictions on publicly sharing data—e.g. participant privacy or use of data from a third party—those must be specified.

Reviewer #1: Yes

Reviewer #2: Yes

**Reply:** Thank you for your comment.

5. Is the manuscript presented in an intelligible fashion and written in standard English?

PLOS ONE does not copyedit accepted manuscripts, so the language in submitted articles must be clear, correct, and unambiguous. Any typographical or grammatical errors should be corrected at revision, so please note any specific errors here.

Reviewer #1: Yes

Reviewer #2: No

**Reply:** Thank you for your comment. Our manuscript has undergone an additional round of English editing to enhance the language and improve clarity and understanding.

6. Review Comments to the Author

Please use the space provided to explain your answers to the questions above. You may also include additional comments for the author, including concerns about dual publication, research ethics, or publication ethics. (Please upload your review as an attachment if it exceeds 20,000 characters)

Reviewer #1: Dear Author
After moderate revision, I have identified two additional suggestions that could still benefit this article from further improvements.

1.Although the introduction has been improved, it could benefit from a clearer statement of the research gap and the novelty of the study. Explicitly highlight how this study differs from previous research and what new insights it offers.

**Reply:** Thank you for your comment. We have included a statement of the research gap on page 5, line 92, to emphasize how our study differs from previous research.

**Page 5, line 92.**

Therefore, body composition indices varied according to sex, and muscle strength was a key measure of lean indices. Bone strength was defined as the integration of bone density and quality. To the best of our knowledge, no previous study has comprehensively investigated the association between different fat and lean indices with bone strength in men and women. Therefore, we aimed to elucidate the effects of the lean and fat indices on bone strength in men and women.

2.Although the discussion section has been expanded, some results, such as the differences in how fat and lean indices contribute to bone strength in men and women, could be interpreted more thoroughly. Discuss the potential biological mechanisms in more detail and relate your findings more explicitly to the existing literatures.

**Reply:** Thank you for your comment. We have discussed the potential biological mechanisms in greater detail on page 17, line 260.

**Page 17, line 260.**

The different contributions of fat and muscle to bone health in men and women are primarily driven by biological mechanisms related to hormonal regulation, mechanical loading, and the distinct ways in which fat and muscle interact with bone tissue. The muscle includes mechanostat function, in which muscles apply stress to bones through contractions during movement, triggering bone remodeling. This mechanical loading is detected by osteocytes, which then signal new bone formation (osteogenesis) to reinforce the skeletal structure [25, 26]. Men typically have greater muscle mass, leading to greater mechanical loading on bones. This results in a stronger stimulus for bone formation and an increased bone mineral density. In contrast, women tend to have a lower muscle mass, which results in less mechanical loading and a weaker stimulus for bone remodeling. This can contribute to relatively lower bone mineral density, particularly after menopause, when muscle mass may further decline. Second, muscles release signaling molecules called myokines that influence bone metabolism. Higher muscle mass in men leads to greater secretion of myokines such as insulin-like growth factor-1, which promotes both muscle growth and bone formation [28, 29]. Although women also benefit from myokine release, their lower muscle mass results in a reduced contribution of this pathway to bone-building processes. The role of fat includes mechanical loading from body weight. Like muscle mass, increased fat mass exerts mechanical loading on bones, stimulating bone formation [11]. Although this effect is less direct and efficient than muscle-induced mechanical loading, body weight from fat still contributes to bone health. Men generally have less body fat than women, leading to less mechanical loading from fat. In contrast, higher body fat in women contributes more significantly to mechanical loading and may positively influence bone mineral density. However, excessive fat can have negative effects, as it may cause systemic inflammation [27]. Second, increased fat-derived hormones (adipokines) such as leptin produced by increased adipose tissue seem to have a protective effect on bone tissue [30]. Leptin plays a less significant role in maintaining bone mineral density in men because of their lower fat mass, whereas it plays a more substantial role in women because of their higher fat mass [31, 32]. The complex interplay between muscle, fat, and hormones affects bone health differently in men and women, with hormonal differences being central to modulating the effect of fat and muscle on bone health.

Reviewer #2: Comments (Reviewer#2)

Thank you for the correction.
After the correction, the issue has been clarified. However, in order to improve the content, we would like to comment.

1, Minor Comment
The previous abstract was presented in the following sections: objectives (e.g. background), methods, results and conclusions, but no distinction is made in the revised version. Was this removed due to special instructions?
If not, it would be better to add the terms purpose (e.g. background), methods, results and conclusions.

**Reply:** Thank you for your comment. The editors requested that our manuscript comply with PLOS ONE's style guidelines, which require an unstructured abstract.

The PLOS ONE style templates can be found at 
<https://journals.plos.org/plosone/s/file?id=wjVg/PLOSOne_formatting_sample_main_body.pdf> and 
<https://journals.plos.org/plosone/s/file?id=ba62/PLOSOne_formatting_sample_title_authors_affiliations.pdf>

2, Major Comment
Concerning the concluding part of the abstract, P2, L37-, and the concluding part of the text, P17, L269-.
The "wording and text in the conclusion of the abstract" does not adequately reflect the "conclusions of the main text". For example, in the conclusion of the main text, men show a relationship between HGS and BMD. However, the abstract conclusion describes a correlation between bone strength in men. The conclusion is the claim of this paper and is very important. The conclusion in the main text and the conclusion in the abstract do not have to be exactly the same sentence, but they should make the same claim.

**Reply:** Thank you for your comment. We have revised the abstract to reflect the same claim found on page 2, line 37.

**Page 2, line 37.**

Fat indices contributed more to bone mineral density in women, whereas lean indices were more positively associated with bone strength in men. Considering different fat indices, appendicular lean mass was more strongly associated with bone mineral density in women, whereas hand grip strength contributed more to bone mineral density in men. In other words, maintaining muscle mass and strength is equally important for bone health.

7. PLOS authors have the option to publish the peer review history of their article ([what does this mean?](https://c05y1x9s.r.us-east-2.awstrack.me/L0/https:%2F%2Fjournals.plos.org%2Fplosone%2Fs%2Feditorial-and-peer-review-process%23loc-peer-review-history/1/010f0191c6320010-e2ccbdd3-2261-43ca-a600-f0521ec8efa1-000000/wTupm_QL-dwMV8O9gWnqtZh6HG0=175)). If published, this will include your full peer review and any attached files.

If you choose “no”, your identity will remain anonymous but your review may still be made public.

**Do you want your identity to be public for this peer review?** For information about this choice, including consent withdrawal, please see our [Privacy Policy](https://c05y1x9s.r.us-east-2.awstrack.me/L0/https:%2F%2Fwww.plos.org%2Fprivacy-policy/1/010f0191c6320010-e2ccbdd3-2261-43ca-a600-f0521ec8efa1-000000/Lcc6jqOo5UTKytsnqab8ZO3cDcc=175).

Reviewer #1: No

Reviewer #2: No

**Reply:** Thank you for your comment.
