# Supplementary material for: Different contributions of fat and lean indices to bone strength by sex
Source: PLoS One. 2024 Nov 14;19(11):e0313740. doi: 10.1371/journal.pone.0313740 (PMC11563455; doi:10.1371/journal.pone.0313740)
Supplement: Attachment — Submitted filename: Response to reviewers 3.docx [file pone.0313740.s003.docx]

**Comments to the Author**

1. If the authors have adequately addressed your comments raised in a previous round of review and you feel that this manuscript is now acceptable for publication, you may indicate that here to bypass the “Comments to the Author” section, enter your conflict of interest statement in the “Confidential to Editor” section, and submit your "Accept" recommendation.

Reviewer #1: All comments have been addressed

Reviewer #2: All comments have been addressed

**Reply:** Thank you for your comment.

2. Is the manuscript technically sound, and do the data support the conclusions?

The manuscript must describe a technically sound piece of scientific research with data that supports the conclusions. Experiments must have been conducted rigorously, with appropriate controls, replication, and sample sizes. The conclusions must be drawn appropriately based on the data presented.

Reviewer #1: Yes

Reviewer #2: Yes

**Reply:** Thank you for your comment.

3. Has the statistical analysis been performed appropriately and rigorously?

Reviewer #1: Yes

Reviewer #2: Yes

**Reply:** Thank you for your comment.

4. Have the authors made all data underlying the findings in their manuscript fully available?

The [PLOS Data policy](http://c05y1x9s.r.us-east-2.awstrack.me/L0/http:%2F%2Fwww.plosone.org%2Fstatic%2Fpolicies.action%23sharing/1/010f019233361042-f42ed944-bcce-4d14-9a30-9fd3179800a6-000000/e2a_0okxVSjgjYMKPor2SjJXA0Q=178) requires authors to make all data underlying the findings described in their manuscript fully available without restriction, with rare exception (please refer to the Data Availability Statement in the manuscript PDF file). The data should be provided as part of the manuscript or its supporting information, or deposited to a public repository. For example, in addition to summary statistics, the data points behind means, medians and variance measures should be available. If there are restrictions on publicly sharing data—e.g. participant privacy or use of data from a third party—those must be specified.

Reviewer #1: Yes

Reviewer #2: Yes

**Reply:** Thank you for your comment.

5. Is the manuscript presented in an intelligible fashion and written in standard English?

PLOS ONE does not copyedit accepted manuscripts, so the language in submitted articles must be clear, correct, and unambiguous. Any typographical or grammatical errors should be corrected at revision, so please note any specific errors here.

Reviewer #1: Yes

Reviewer #2: Yes

**Reply:** Thank you for your comment.

6. Review Comments to the Author

Please use the space provided to explain your answers to the questions above. You may also include additional comments for the author, including concerns about dual publication, research ethics, or publication ethics. (Please upload your review as an attachment if it exceeds 20,000 characters)

Reviewer #1: Dear Author
The author revised the abstract to align with the main text, ensuring consistency in the conclusions drawn about handgrip strength (HGS) and bone mineral density (BMD) between men and women. The inclusion of a more detailed explanation about the different contributions of fat and lean indices also provides greater clarity to the biological mechanisms behind the sex differences in bone strength
After 2nd revision, there are still minor points worth noting:

1.While the revised abstract now mirrors the conclusions in the text, it could still benefit from being more concise. The distinction between fat indices and lean indices, as well as their contributions to bone health, is now clearer, but simplifying the language in the abstract would improve readability.

**Reply:** Thank you for your comment. The abstract has undergone an additional round of English editing to enhance the language and improve clarity and understanding.

**Page 2, line 20.**

**ABSTRACT**

Bone strength depends on both bone density and quality. However, the impact of body composition indices on bone strength in men and women remains unclear. This study aimed to investigate the association of different fat and lean indices with bone strength according to sex. In this cross-sectional study involving 1,419 participants, bone mineral density (BMD) and body composition were measured using dual-energy X-ray absorptiometry. Bone quality was assessed using the trabecular bone score (TBS). Fat indices included total fat mass, body fat percentage, and waist circumference, while lean indices included appendicular lean mass (ALM) and hand grip strength. All fat indices demonstrated a positive association with BMD and a negative association with TBS in both men and women. Fat indices were more closely associated with BMD in women than in men. Furthermore, lean indices contributed more to BMD in men than in women. In women, ALM contributed more to BMD than hand grip strength, whereas in men, hand grip strength had a greater impact on BMD than ALM. Hand grip strength was also positively associated with TBS in men. Overall, fat indices had a greater influence on BMD in women, while lean indices were more positively associated with bone strength in men. Considering different fat indices, ALM was more closely associated with BMD in women, whereas hand grip strength played a greater role in men. Thus, maintaining both muscle mass and strength is crucial for preserving bone mass.

2.The expanded discussion about potential biological mechanisms is comprehensive, but a more focused discussion on the most relevant mechanisms could streamline this section.

**Reply:** Thank you for your comment. We have focused on the most relevant mechanisms in the discussion section on page 17, line 260.

**Page 17, line 260.**

The different contributions of fat and muscle to bone health in men and women are primarily driven by biological mechanisms related to mechanical loading and the distinct ways in which fat and muscle interact with bone tissue. The muscle includes mechanostat function, in which muscles apply stress to bones through contractions during movement, triggering bone remodeling. This mechanical loading is detected by osteocytes, which then signal new bone formation (osteogenesis) to reinforce the skeletal structure [25, 26]. Men typically have greater muscle mass, leading to greater mechanical loading on bones. This results in a stronger stimulus for bone formation and an increased BMD. In contrast, women tend to have a lower muscle mass, which results in less mechanical loading and a weaker stimulus for bone remodeling. This can contribute to relatively lower BMD, particularly after menopause, when muscle mass may further decline. The role of fat includes mechanical loading from body weight. Like muscle mass, increased fat mass exerts mechanical loading on bones, stimulating bone formation [11]. Although this effect is less direct and efficient than muscle-induced mechanical loading, body weight from fat still contributes to bone health. Men generally have less body fat than women, leading to less mechanical loading from fat. In contrast, higher body fat in women contributes more significantly to mechanical loading and may positively influence BMD. However, excessive fat can have negative effects, as it may cause systemic inflammation [27]. The complex interplay between muscle and fat affects bone health differently in men and women, with hormonal differences modulating the effect of fat and muscle on bone health [25, 26].

Reviewer #2: (No Response)

**Reply:** Thank you for your comment.

7. PLOS authors have the option to publish the peer review history of their article ([what does this mean?](https://c05y1x9s.r.us-east-2.awstrack.me/L0/https:%2F%2Fjournals.plos.org%2Fplosone%2Fs%2Feditorial-and-peer-review-process%23loc-peer-review-history/1/010f019233361042-f42ed944-bcce-4d14-9a30-9fd3179800a6-000000/r6blPxyxc86JW7odLw_B92QOzok=178)). If published, this will include your full peer review and any attached files.

**Do you want your identity to be public for this peer review?** For information about this choice, including consent withdrawal, please see our [Privacy Policy](https://c05y1x9s.r.us-east-2.awstrack.me/L0/https:%2F%2Fwww.plos.org%2Fprivacy-policy/1/010f019233361042-f42ed944-bcce-4d14-9a30-9fd3179800a6-000000/wonRPm0RolYkgGxxlrD4d_pGXrc=178).

Reviewer #1: **Yes:** Robert Wen-Wei Hsu

Reviewer #2: No

**Reply:** Thank you for your comment.
