# Supplementary material for: Different contributions of fat and lean indices to bone strength by sex
Source: PLoS One. 2024 Nov 14;19(11):e0313740. doi: 10.1371/journal.pone.0313740 (PMC11563455; doi:10.1371/journal.pone.0313740)
Supplement: Attachment — Submitted filename: Response to reviewers 4.docx [file pone.0313740.s004.docx]

**Comments to the Author**

1. If the authors have adequately addressed your comments raised in a previous round of review and you feel that this manuscript is now acceptable for publication, you may indicate that here to bypass the “Comments to the Author” section, enter your conflict of interest statement in the “Confidential to Editor” section, and submit your "Accept" recommendation.

Reviewer #1: All comments have been addressed

Reviewer #3: All comments have been addressed

**Reply:** Thank you for your comment.

2. Is the manuscript technically sound, and do the data support the conclusions?

The manuscript must describe a technically sound piece of scientific research with data that supports the conclusions. Experiments must have been conducted rigorously, with appropriate controls, replication, and sample sizes. The conclusions must be drawn appropriately based on the data presented.

Reviewer #1: Yes

Reviewer #3: Yes

**Reply:** Thank you for your comment.

3. Has the statistical analysis been performed appropriately and rigorously?

Reviewer #1: Yes

Reviewer #3: Yes

**Reply:** Thank you for your comment.

4. Have the authors made all data underlying the findings in their manuscript fully available?

The [PLOS Data policy](http://c05y1x9s.r.us-east-2.awstrack.me/L0/http:%2F%2Fwww.plosone.org%2Fstatic%2Fpolicies.action%23sharing/1/010f0192b9b9c574-d4143918-8f28-4599-bcf3-8a729cad0d8b-000000/zT6QMCvBErbECsuLeDb7WjbkrZM=182) requires authors to make all data underlying the findings described in their manuscript fully available without restriction, with rare exception (please refer to the Data Availability Statement in the manuscript PDF file). The data should be provided as part of the manuscript or its supporting information, or deposited to a public repository. For example, in addition to summary statistics, the data points behind means, medians and variance measures should be available. If there are restrictions on publicly sharing data—e.g. participant privacy or use of data from a third party—those must be specified.

Reviewer #1: Yes

Reviewer #3: Yes

**Reply:** Thank you for your comment.

5. Is the manuscript presented in an intelligible fashion and written in standard English?

PLOS ONE does not copyedit accepted manuscripts, so the language in submitted articles must be clear, correct, and unambiguous. Any typographical or grammatical errors should be corrected at revision, so please note any specific errors here.

Reviewer #1: Yes

Reviewer #3: Yes

**Reply:** Thank you for your comment.

6. Review Comments to the Author

Please use the space provided to explain your answers to the questions above. You may also include additional comments for the author, including concerns about dual publication, research ethics, or publication ethics. (Please upload your review as an attachment if it exceeds 20,000 characters)

Reviewer #1: Dear Author:
The authors addressed key concerns by aligning the abstract with the findings, refining biological mechanism discussions, and checking for multicollinearity. However, further streamlining of the abstract and organization of the discussion would enhance clarity. A post-hoc power analysis and exploration of interaction effects are still needed to strengthen statistical rigor.
The authors appropriately acknowledge limited generalizability and suggest future studies. With minor revisions—focusing on clarity, statistical robustness, and data availability—the manuscript will be ready for publication and provide valuable insights into bone health differences across sexes.

**Reply:** We appreciate your valuable comments to help improve the quality of our manuscript. In response, we have not only conducted an additional round of English editing to enhance clarity and comprehension, but also strengthened the statistical robustness of our analysis on page 8, line 151 and page 12, line 193. Our responses to the reviewers' comments are provided below, and the revised manuscript has been updated accordingly. Additionally, the dataset is owned by the IRB of Cheng Hsin General Hospital. The IRB approved the data analysis for our study but did not approve data sharing. Therefore, we do not have permission to share the dataset. Interested researchers can request access to the data by contacting the IRB of Cheng Hsin General Hospital at chghirb@chgh.org.tw. Access to the data will be granted in the same manner as it was for the authors, who do not have any special access privileges. We have included the data availability statement on page 20, line 317. We hope that the revised manuscript will have an enhanced scientific impact.

**Page 8, line 151**

An interaction model was built to investigate the statistical interaction between body composition indices and sex. A post-hoc power analysis based on an alpha level (0.05) was performed to strengthen the statistical robustness.

**Page 12, line 187**

HGS was more positively associated with BMDs and the TBS in men than in women. In contrast, HGS showed minimal association with total hip BMD in women (Table 3). Additionally, there was significant interaction between HGS and sex. The VIFs in all models were less than 10, indicating a low degree of multicollinearity. As with women, the R^2^ values in men for spine BMD and the TBS were low, but they were high for both femoral neck and total hip BMDs. In contrast, when models were adjusted for HGS, the R^2^ values were higher in men than in women. A post-hoc power analysis showed that the power exceeded 80%.

**Page 18, line 290**

Nevertheless, our study had some limitations that must be considered when interpreting the results. First, this was a cross-sectional study; therefore, the causal inferences could not be made. Second, the participants belonged exclusively to the Asian population, which may have affected the generalizability of our results. Third, our study only included men aged ≥50 years and post-menopausal women; therefore, our results may not be applicable to younger men or premenopausal women. Further studies across various populations were needed to confirm our results.

**Page 20, line 317**

**Data Availability statement:**

The data set is owned by the institutional review board of Cheng Hsin General Hospital. The institutional review board of Cheng Hsin General Hospital only approved the data analysis in our study and did not approve data sharing. Therefore, we do not have permission to share the data set. Interested researchers can submit data access requests to the institutional review board of Cheng Hsin General Hospital through the following email address: chghirb@chgh.org.tw Others would be able to access the data in the same manner as the authors.

Reviewer #3: Osteoporosis is recognized as a serious problem that is associated with the need for nursing care and threatens healthy life expectancy. This study can be applied to simple prediction of regional BMD related to fractures in adults in the Asian region. It is expected that this method will be used in primary care in the future. The review comments to date have been fully addressed.

**Reply:** Thank you for your comment.

7. PLOS authors have the option to publish the peer review history of their article ([what does this mean?](https://c05y1x9s.r.us-east-2.awstrack.me/L0/https:%2F%2Fjournals.plos.org%2Fplosone%2Fs%2Feditorial-and-peer-review-process%23loc-peer-review-history/1/010f0192b9b9c574-d4143918-8f28-4599-bcf3-8a729cad0d8b-000000/Ymn8RLiQRoRJYTP9u9kh4cKBvKg=182)). If published, this will include your full peer review and any attached files.

If you choose “no”, your identity will remain anonymous but your review may still be made public.

**Do you want your identity to be public for this peer review?** For information about this choice, including consent withdrawal, please see our [Privacy Policy](https://c05y1x9s.r.us-east-2.awstrack.me/L0/https:%2F%2Fwww.plos.org%2Fprivacy-policy/1/010f0192b9b9c574-d4143918-8f28-4599-bcf3-8a729cad0d8b-000000/p8_RIjsTKSZvHWsyHirDEUdVMwU=182).

Reviewer #1: **Yes:** Robert Wen-Wei Hsu

Reviewer #3: No

**Reply:** Thank you for your comment.
